# An Engineering Alternative to Lockdown During COVID-19 and Other Airborne Infectious Disease Pandemics: Feasibility Study

**DOI:** 10.2196/54666

**Published:** 2024-05-14

**Authors:** Yusaku Fujii

**Affiliations:** 1 School of Science and Technology Gunma University Kiryu Japan

**Keywords:** COVID-19, airborne infectious diseases, lockdown, powered air purifying respirator (PAPR), infectious dose, airborne transmission, emergency evacuation, herd immunity, pandemic, aerosol, air, quality, infection control, infectious, respiratory, purifier, purifiers, purifying, respirator, respirators, device, devices, airborne

## Abstract

**Background:**

Now and in the future, airborne diseases such as COVID-19 could become uncontrollable and lead the world into lockdowns. Finding alternatives to lockdowns, which limit individual freedoms and cause enormous economic losses, is critical.

**Objective:**

The purpose of this study was to assess the feasibility of achieving a society or a nation that does not require lockdown during a pandemic due to airborne infectious diseases through the mass production and distribution of high-performance, low-cost, and comfortable powered air purifying respirators (PAPRs).

**Methods:**

The feasibility of a social system using PAPR as an alternative to lockdown was examined from the following perspectives: first, what PAPRs can do as an alternative to lockdown; second, how to operate a social system utilizing PAPR; third, directions of improvement of PAPR as an alternative to lockdown; and finally, balancing between efficiency of infection control and personal freedom through the use of Internet of Things (IoT).

**Results:**

PAPR was shown to be a possible alternative to lockdown through the reduction of airborne and droplet transmissions and through a temporary reduction of infection probability per contact. A social system in which individual constraints imposed by lockdown are replaced by PAPRs was proposed, and an example of its operation is presented in this paper. For example, the government determines the type and intensity of the lockdown and activates it. At that time, the government will also indicate how PAPR can be substituted for the different activity and movement restrictions imposed during a lockdown, for example, a curfew order may be replaced with the permission to go outside if wearing a PAPR. The following 7 points were raised as directions for improvement of PAPR as an alternative method to lockdown: flow optimization, precise differential pressure control, design improvement, maintenance method, variation development such as booth type, information terminal function, and performance evaluation method. In order to achieve the effectiveness and efficiency in controlling the spread of infection and the individual freedom at a high level in a social system that uses PAPRs as an alternative to lockdown, it was considered effective to develop a PAPR wearing rate network management system utilizing IoT.

**Conclusions:**

This study shows that using PAPR with infection control ability and with less economic and social damage as an alternative to nationwide lockdown is possible during a pandemic due to airborne infectious diseases. Further, the efficiency of the government’s infection control and each citizen’s freedom can be balanced by using the PAPR wearing rate network management system utilizing an IoT system.

## Introduction

For more than 3 years, herd immunity has been pursued worldwide through vaccination as a countermeasure against COVID-19, in addition to new lifestyle measures (social distancing, wearing of face masks, washing of hands, etc) [1,2]. However, due to the emergence of new variants, the spread of infection has sometimes been uncontrollable in different parts of the world. Each time, lockdown has been implemented to temporarily buy time, causing great economic loss and restriction of freedom for individuals, businesses, and society [3-7]. In this study, lockdown is defined as restricting the actions and activities of people and businesses to temporarily slow the spread of infection and buy time for other measures such as herd immunity through vaccination. A strong lockdown includes orders with criminal penalties that prohibit going out, working, and doing business, and a weak lockdown includes voluntary requests to refrain from going out, working, and doing business. The type and strength of lockdowns are determined by the government on a case-by-case basis, considering the impact on infection control and harm to society, depending on the situation of the outbreak of infection at the time [8,9].

Even in the current situation, the rapid development and delivery of effective vaccines against new variants of the coronavirus (SARS-CoV-2) and new airborne viruses that may emerge in succession is not well assured. In the future, it is likely that we will continue to be in a situation where we do not know when a lockdown will again be necessary around the world [10,11]. In light of this, alternatives to lockdown that can cause less economic damage, avoid restrictions on freedom of action, and other disadvantages to individuals, companies, and societies would be beneficial. Among the modes of COVID-19 transmission, contact and oral infections are relatively easy to prevent by environmental hygiene, including handwashing and food hygiene management. Droplet infection (particle size ≥100 µm) is thought to be preventable by social distancing (droplets fall by gravity) and by wearing a mask. Currently, airborne transmission via aerosols (particle size <100 µm) is thought to be the main route of infection [12,13]. Although the infectious dose of COVID-19, that is, the number of ingested viruses required for infection, is not well known [14], it is estimated to be in the range of 300 to 2000 virions [15]. It is believed that the possibility of viral infection can be effectively reduced by shielding aerosols that may contain viruses. This paper discusses alternative means to lockdown, assuming that contact and oral infections are prevented by environmental hygiene and food hygiene management and that only airborne and droplet infections remain as infection routes.

## Methods

The feasibility of a social system utilizing powered air purifying respirators (PAPRs) as an alternative to lockdown was examined from the following perspectives: (1) what PAPR can do as an alternative to lockdown, (2) how to operate a social system utilizing PAPR, (3) direction of improvement of PAPR as an alternative to lockdown, and (4) balance between efficiency of infection control and personal freedom through the use of Internet of Things (IoT).

### What PAPR Can Do as an Alternative to Lockdown

The PAPR for practical medical use is a device that drastically reduces the number of viruses inhaled by the wearer and effectively reduces the risk of infection. Medical PAPRs are used by medical personnel working in high-risk environments [16,17]. The assigned protection factor (APF), as defined by the National Institute for Occupational Safety and Health in the United States, is widely used as an indicator of the shielding performance of respiratory protection devices, including PAPRs [18]. APF is defined as the external concentration/internal concentration of the target particles (aerosols). For a medical face mask, APF=10 is given when a person who has been trained to wear it wears it completely with no gaps between the mask and the facial surface. However, 3M PAPR is rated at APF=1000 and is considered to have excellent protective performance [19]. In other words, the concentration of virus-containing aerosols can be reduced to 1/10 or less with a full-face mask when worn without gaps, while it is reduced to 1/1000 or less with 3M PAPR. Therefore, high-performance PAPRs can be used as an alternative to the movement restrictions and activity restrictions imposed by lockdowns used as a countermeasure against COVID-19 and other airborne infectious diseases in future pandemics. The necessary conditions for an alternative to lockdown are that it should have the same deterrent effect on the spread of infection as lockdown, and the economic damage, activity restrictions, and other disadvantages to individuals and society should be smaller compared to those during lockdown.

The effective reproduction number R_t_ is the average number of secondary cases per infectious case in a population of both susceptible and nonsusceptible individuals. In this population, R_t_<1 means converging, R_t_=1 means stationary, and R_t_>1 means expanding [20]. The effective reproduction number R_t_ can be expressed schematically by the following equation.

R_t_ = β × k × D, where β=probability of infection being transmitted during a contact, k (contact/day)=contact rate in the host population, and D (day)=duration of infectiousness.

**Figure 1:**
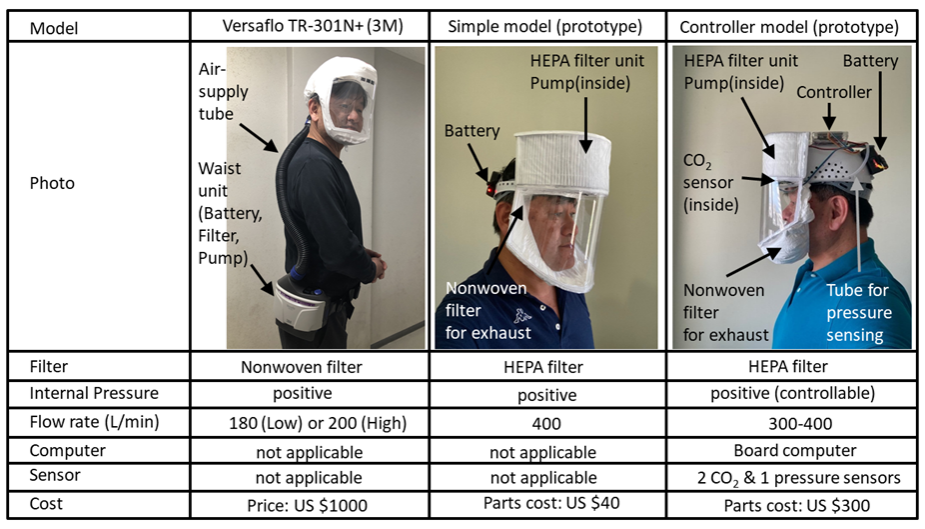
Specifications of the 3 existing powered air purifying respirators. HEPA: high efficiency particulate air.

To control the infection of the whole society, it is sufficient to set the effective reproduction number (R_t_) to <1. To do this, we should reduce the above β, k, and D. Vaccines are expected to reduce β in the long term. Lockdown is expected to play a role in temporarily reducing k. In this study, PAPR is expected to play a role in temporarily reducing β by reducing the airborne and droplet transmission. During COVID-19, the maximum R_t_ value reported worldwide was around 5 [21,22]. If lockdown is used as a means of correcting R_t_=5 to R_t_=1, it is sufficient if the lockdown makes k 1/5 of its current value. Alternatively, if PAPRs are used as an alternative to lockdown, it would be sufficient if citizens could wear PAPRs to reduce β to 1/5 of its current value. It is worth noting here that the degree of reduction in k and β may be about 1/5 rather than 1/100 or 1/1000. The performance of the existing PAPRs was evaluated. Figure 1 shows the specifications of 3 existing PAPRs.

### Currently Existing PAPRs

#### Medical PAPR: Versaflo TR-301N+

This PAPR is marketed as a medical PAPR. The waist unit (model TR-301N+; 3M Corp) contains a nonwoven fabric filter (model TR-3712N), pump, and battery (model TR-332). Purified air is pumped from the waist unit into the hood (model S-133L) through a flexible hose (model PSD-0225). The outline of this PAPR is as follows. Only air purified by a high-performance nonwoven filter is introduced into the hood by a pump. Since positive pressure is naturally maintained inside the hood, outside air is prevented from entering even if there is a gap between the hood seal and the face and the head. Thus, the air, which the wearer breathes, is only the air purified by the nonwoven filter. As for the face mask, since the inside of the mask becomes negative pressure during inhalation, if there is a gap between the face and the mask, the outside air will enter directly through the gap during the wearer’s inhalation [23,24]. Therefore, PAPR is structurally capable of shielding most of the aerosols present in the ambient air. This is supported by the fact that this PAPR has a high value of APF=1000. The flow rate can be selected in 2 stages, that is, high and low, and the specified flow rates are approximately 6.5 cubic feet per minute (180 L/min) and 7.2 cubic feet per minute (200 L/min), respectively [25].

#### PAPR Prototype With a Simple Structure

This PAPR is a prototype developed as a low-cost PAPR that has a simple structure similar to the abovementioned commercially available medical PAPR. The specifications of the air purification characteristics in this prototype are as follows [26].

1. Only air purified by a high-efficiency nonwoven filter is pumped into the hood.

2. Because positive pressure is naturally maintained inside the hood, outside air is prevented from entering even if there is a gap between the hood seal and the face.

3. The exhaust is natural exhaust from a thin nonwoven fabric filter due to the positive pressure inside the hood. Even if the wearer is infected and emits droplets or aerosols containing the virus, it is possible to prevent some of the external emissions.

The first 2 characteristics mentioned above are the same as those for the medical PAPR mentioned above (TR-301N+). In the air supply side, a nonwoven fabric filter—high-efficiency particulate air filter—which can filter 99.97% or more of aerosols down to 0.3 µm or larger is used on the air supply side. For aerosols containing viruses, it is considered sufficient to target aerosols with a particle size of 0.3 µm or larger [27,28]. A high-performance PAPR can be assembled at a total parts cost of approximately US $40 with a simple configuration of only a high performance filter, battery, and pump.

#### PAPR Prototype With a Controller

This PAPR is a prototype developed as a high-performance PAPR equipped with a controller/computer for measurement and control [29]. The specifications for the air purification characteristics are the same as those in the PAPR prototype with a simple structure. In addition to the characteristics of the PAPR prototype with a simple structure, a controller (on-board computer) and sensors (2 CO_2_ concentration sensors and 1 differential pressure sensor) are added, and the pump is controlled by means of the pulse width modulation control. In this PAPR, the pump output is adjusted and controlled according to the output of the differential pressure sensor so as to suppress the internal pressure fluctuations due to breathing, that is, higher pressure during exhalation and lower pressure during inspiration. Operating parameters can be set and monitored using a smartphone. It is also possible to connect to the internet via the smartphone. As a result, it is possible to connect to the PAPR wearing rate network management system. In addition, by installing a pump and filter on the exhaust side and making it a differential type, it is possible to set the internal pressure to either positive pressure or negative pressure. Thus, it is possible to set the positive pressure setting to protect the wearer from the outside and to set the negative pressure setting to protect the outside from the wearer. It is possible to manufacture this PAPR with a total parts cost of approximately US $300; this PAPR allows pump control based on sensor signals and settings and monitoring of the operating parameters by using a smartphone.

### How to Operate a Social System Utilizing PAPR

A case in which a fixed percentage of the population wears PAPRs is considered. Quantitative evaluation of the reduction in the aforementioned β (infection probability per contact) by wearing PAPR and quantitative evaluation of the reduction in R_t_ by wearing PAPR are considered to require empirical and social experiments, as described below, because they involve humans. However, as shown below, quantitative evaluation is possible under limited conditions. Assuming that the aerosols and droplet shielding rate by PAPRs is the same for all particle sizes of aerosols and droplets, the shielding rate can be expressed as follows: (1) shielding rate for aerosols and droplets in the air supply (R_S,in_) and (2) shielding rate for aerosols and droplets in the exhaust air (R_S,ex_). Assume that the reduction rate of the probability of the wearer himself/herself becoming infected and the reduction rate of the probability of the wearer infecting others by wearing PAPR are as follows: (3) reduction rate of the probability that the wearer will be infected by aerosols and droplets in the air supply (R_I,in_) and (4) reduction rate of the probability of infecting others by aerosols and droplets in exhaust air (R_I,ex_). It is difficult to quantitatively determine the relationship between (1) and (3) and between (2) and (4) above. However, the following limited arrangement can be made. For the air supply side, the following can be said.

1. If the aerosol and droplet shielding ratio of PAPR is perfect (R_S,in_=1), wearing PAPR will reduce the probability of infection by means of aerosols and droplets in the supply air by 100% (R_I,i_=1).

2. If the aerosol and droplet shielding ratio of PAPR is nothing (R_S,in_=0), wearing PAPR will reduce the probability of infection by means of aerosols and droplets in the supply air by nothing (R_I,in_=0).

3. In the intervals of 0<R_S,in_<1 and 0<R_I,in_<1, there is a positive correlation between R_S,in_ and R_I,in_.

For the exhaust side, the following can be said.

4. If the aerosol and droplet shielding ratio of PAPR is perfect (R_S,ex_=1), wearing PAPR will reduce the probability of infecting others by means of the aerosols and droplets in the exhaust air by 100% (R_I,ex_=1).

5. If the aerosol and droplet shielding ratio of PAPR is nothing (R_S,ex_=0), wearing PAPR will reduce the probability of infecting others by means of the aerosols and droplets in the exhaust air by nothing (R_I,ex_=0).

6. In the intervals of 0<R_S,ex_<1 and 0<R_I,ex_<1, there is a positive correlation between R_S,ex_ and R_I,ex_.

A social group was assumed to be completely free from contact and oral infections, and airborne and droplet infections were the only routes of infection. It is assumed that a certain percentage of the population in the social group always wears a PAPR. The performance of PAPR is assumed to be as follows.

1. The shielding rate of aerosols and droplets in the air supply side is 100% (R_S,in_=1). As a result, the reduction rate of the probability of infection by aerosol and droplets in the air supply is 100% (R_I,in_=1).

2. The shielding rate of aerosols and droplets in the exhaust side is the same as that of the face mask used in the population at that time.

3. When the above PAPR is worn by a percentage of people in W_R_, the following relationship is established between the effective reproduction number R_t_ immediately before the start of wearing and the modified effective reproduction number R_tm_ immediately after the start of wearing.

R_tm_ = [0.0 W_R_ + 1.0 (1–W_R_)] R_t_ = (1–W_R_) R_t_

The expression for W_R_ is as follows.

W_R_ = 1–R_tm_ / R_t_

**Figure 2:**
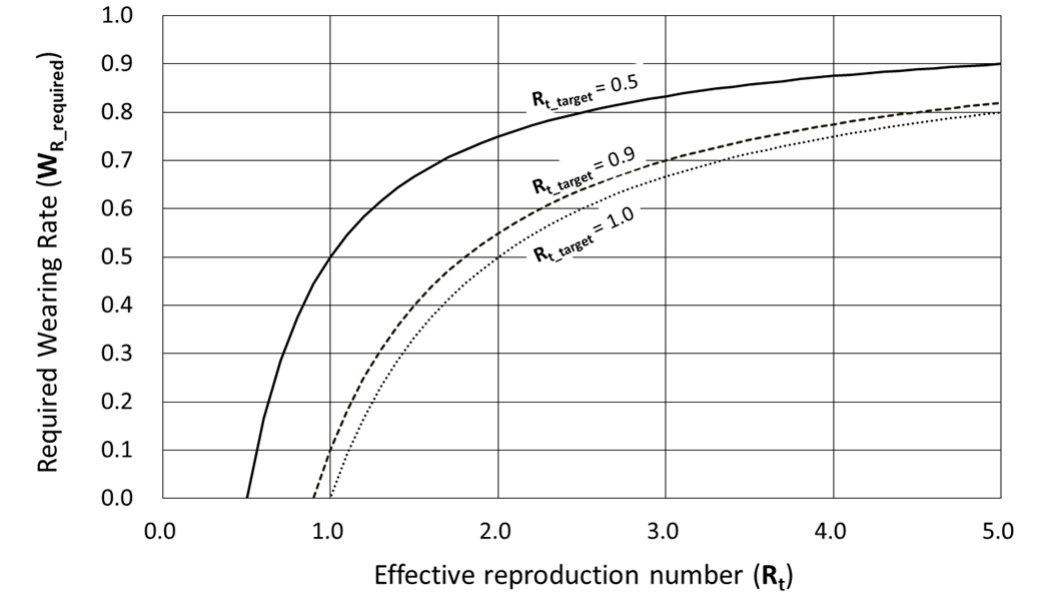
Relationship between the effective reproduction number and the required wearing rate, which is required to achieve the target effective reproduction number of 0.5, 0.9, and 1.0. R_t_target_: target effective reproduction number.

Figure 2 shows the relationship between the effective reproduction number R_t_ at the time in question and the required wearing rate W_R_required_, which is required to achieve the target effective reproduction number R_tm_ of 0.5, 0.9 and 1.0. For example, consider an event in which a certain percentage of the population is wearing PAPR at all the time. As an example of a situation of severe infection spread, consider the case where R_t_=2 immediately before the start of the event. In this case, to achieve the target effective reproduction number R_t_target_ of 1.0, 0.9, and 0.5, 50%, 55%, and 75% of the population should wear PAPRs at all times, respectively. The above simulation targets a social group in which airborne and droplet infections are the only routes of infection and an extreme setting in which PAPR is worn at all times. Future studies should consider more realistic settings that suit the conditions of daily life. For example, the situations in which the effects of not wearing PAPR should be considered, including contact with family members in the home, eating, drinking, washing, and bathing.

### Direction of Improvement of PAPR as an Alternative to Lockdown

As the directions of improvements of PAPR as an alternative to lockdown, the following 7 points are proposed and discussed: (1) flow path optimization, (2) precise pressure control by fluid modeling, (3) improved design, (4) maintenance method, (5) variations suitable for different places of use and activity contents, (6) PAPR with information terminal function, and (7) evaluation indicators and evaluation methods.

### Balance Between Efficiency of Infection Control and Personal Freedom Through the Use of IoT

**Figure 3:**
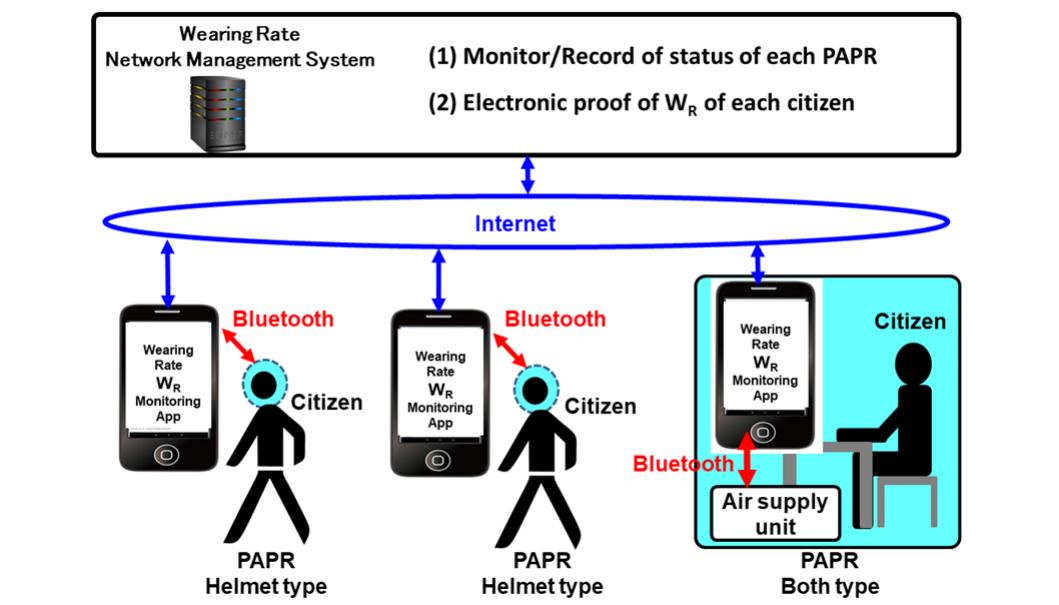
The powered air purifying respirator wearing rate network management system. PAPR: powered air purifying respirator; W_R_: wearing rate.

In order to achieve both (1) effectiveness and efficiency in controlling the spread of infection and (2) individual freedom (limiting the obligation to wear PAPRs to the minimum necessary) at a high level in a social system that uses PAPRs as an alternative to lockdown, it is considered effective to develop a PAPR wearing rate network management system as shown in Figure 3.

1. PAPR (helmet type, booth type, etc) is connected to the wearer’s smartphone via Bluetooth.

2. The smartphone is connected to the internet and connected to the PAPR wearing rate network management system server operated by the government.

3. The government will be able to monitor, record, and manage each citizen’s PAPR wearing rate along with smartphone location information by using the system.

Wearers (citizens) can display the electronic proof of their wearing rate on their smartphones provided by the system. Various parameters can be considered for the PAPR wearing rate (W_R_). As simple examples, the following definitions of wearing rate (W_R_) can be considered.

W_R_=time spent outside with PAPR/time spent outside

Instead of the PAPR wearing rate W_R_, time spent outside without PAPR T_WT_ can be considered.

T_WT_=time spent outside without PAPR.

Instead of the PAPR wearing rate W_R_, the number of viruses inhaled during an outing I_V_ (virions), which is considered to have a direct correlation with infection, could be used as a parameter for evaluation. If the estimated viral concentration d (virions/m^3^) in the activity range is available, the following definition can be adopted.

I_V_=number of viruses inhaled during outings (virions)

=∫d (1–R_S,in_) Q_breath,in_ dt

where, d (virions/m^3^)=estimated viral concentration at the location, R_S,in_=aerosol shielding ratio for the air supply of PAPR, and Q_breath,in_ (m^3^/s)=estimated amount of inhaled air of the wearer at the time (exhaled air is not counted). As a request from the government to each citizen, it is assumed that keeping the above W_R_, T_WT_, or I_V_ at a certain level or better will be requested.

### Ethical Considerations

This study is based on known facts and the author's own thinking, and no new experiments were performed. Therefore, the author has not applied to Gunma University, to which the author belongs, for ethics approval. Consent for publication has been granted from the identifiable individual (author YF) in Figure 1 in this paper.

## Results

### What PAPR Can Do as an Alternative to Lockdown

PAPR was shown to be a possible alternative to lockdown through the reduction of airborne and droplet transmissions and through the temporary reduction of β. The existing medical PAPRs appear to have sufficiently high virus shielding performance and appear to have already reached a level that should be experimentally tested as an alternative to lockdown. The current medical PAPR shown in Figure 1 is expensive and does not have measurement control functions. However, the prototypes shown in Figure 1 indicate that cost reduction and high functionality are possible. In addition, a variety of PAPRs are commercially available for nonmedical use, some of which are inexpensive. If an inexpensive PAPR is supplied to everyone, using PAPRs during a pandemic instead of issuing a countrywide lockdown will become a reality.

### How to Operate a Social System Utilizing PAPR

A realistic process is shown below for quantitatively evaluating the effect of the aerosol shielding performance of PAPRs (for air intake side and exhaust side), PAPR wearing rate and wearing condition for reducing the β, and the effective reproduction number R_t_ for realizing this proposal.

1. Select and prepare special experimental zones for social experiments in the next pandemic.

2. In the experimental zone, when lockdown is applied to the surrounding area, PAPR can be substituted for the various activity restrictions during lockdown.

3. Compare the spread of infection between the special experimental zone and other areas, and change the aerosol shielding performance of PAPR (air supply side and exhaust side), PAPR wearing rate and condition, and other operational conditions within the special experimental zone. The obtained results can be used to quantitatively evaluate the effects of the aerosol shielding performance of PAPR (air supply side and exhaust side), PAPR wearing rate and wearing condition for reducing the β, and the effective reproduction number R_t_.

4. When the effectiveness of PAPR as an alternative to lockdown is confirmed and the problems are sufficiently resolved, PAPR as an alternative to lockdown can be applied to other regions.

**Figure 4:**
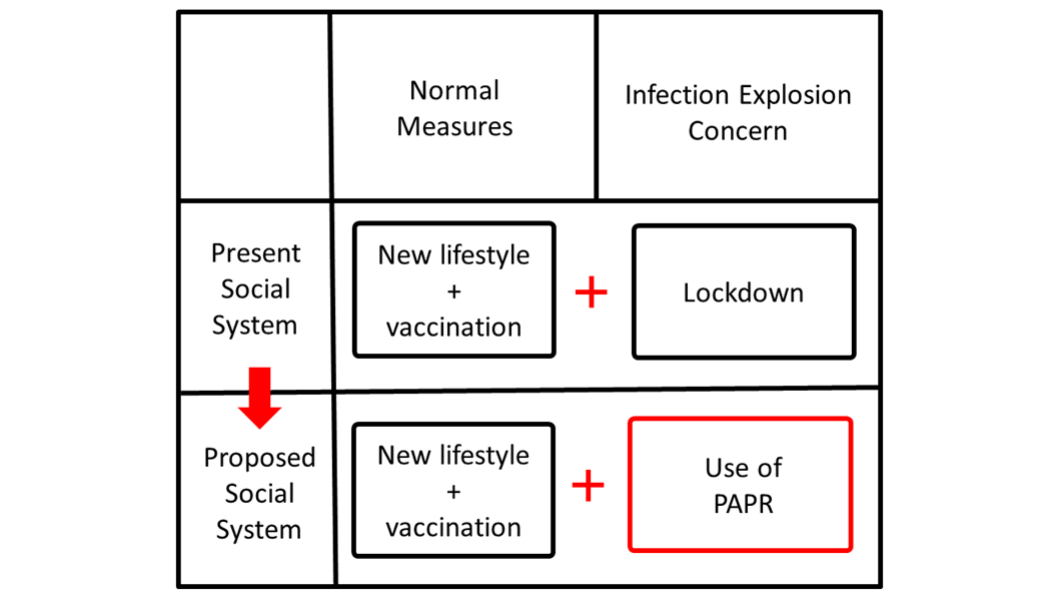
Proposed social system where powered air purifying respirators are used as an alternative to lockdown. PAPR: powered air purifying respirator.

Examples of operations in special experimental zones include the following: (1) people can go out freely if they wear PAPRs, even in circumstances where going out is restricted in other surrounding areas and (2) factories can be operated freely if its employees wear PAPRs, even in circumstances where factories are prohibited to operate in other surrounding areas. Figure 4 shows the proposed social system where PAPRs are used as an alternative to lockdown.

In the proposal in this research, PAPR will be utilized under the leadership of the government as described below:

1. The government distributes PAPRs (helmet/hood type) to all citizens as emergency equipment.

2. If the estimated effective reproduction number R_t_ is high and there is concern about an outbreak of infection, the government will determine the type and intensity of the lockdown and decide how to replace each constraint during lockdown with PAPR. Examples include prohibition on going outside (going outside is possible if wearing a PAPR), prohibition of factory operation (operation is possible if all employees wear PAPRs), prohibition of restaurant operation (operation is possible if all employees wear PAPRs and all customers wear PAPRs suitable for eating and drinking), and overseas entry prohibition (entry is allowed if visitors agree to wear a negative pressure PAPR for a specified period of time and accept government remote monitoring of wearing conditions). This will make it possible to open the door to foreigners and returnees who wish to enter the country, although they would be subject to the same level of inconvenience as ordinary citizens suspected of being infected.

In the initial implementation of the proposed social system, as described above, special experimental zones will be established in various regions, various trials will be conducted based on various assumptions, and data will be collected. Based on the data obtained, qualitative and quantitative evaluations of the benefits (reduction of infection probability) and burdens borne by individuals and the benefits (reduction of infection spread) and burdens for the society as a whole will be attempted. The proposed social system should be compared and verified with the lockdown in each of the different situations, and the best way to be found as an alternative to the various restrictions imposed by the lockdown should be identified. Ultimately, a PAPR-utilizing social system will be constructed that effectively functions as an alternative to lockdown.

### Directions for Improvement of PAPRs as an Alternative to Lockdown

The following 7 points can be considered as directions for improvements of PAPRs as a lockdown alternative.

#### Flow Path Optimization

A hood shape and part configuration should be developed that provides a smooth flow of the exhaled air out of the hood. The concentration of carbon dioxide is approximately 500 ppm (0.05%) in ambient air and approximately 50,000 ppm (5%) in exhaled air [30]. The oxygen concentration in the exhaled air is expected to decrease from the oxygen concentration in the ambient air (approximately 21%) by an amount equal to the increase in the carbon dioxide concentration in the exhaled air (approximately 5%). In the commercially available PAPR and the developed PAPRs shown in Figure 1, a large flow rate (approximately 200-400 L/min) is delivered compared to the resting respiratory flow rate (approximately 6-10 L/min) [31] in order to suppress the carbon dioxide concentration in the hood [25,26,29]. Efficient expiration of exhaled air to the outside allows for a significant reduction in the air supply flow rate, resulting in a significant reduction in the size and weight of pumps, batteries, and filters, as well as design diversification. By minimizing the volume inside the mask, it is also possible to minimize the retention of the exhaled air from the nose and mouth inside the mask. As an extreme example, consider a configuration in which the nose is used for inhalation, the mouth is used for exhalation, and the air supply to the nose and the exhaust from the mouth are mechanically separated. As a result, the flow rate of the air filtered through the nonwoven filter and delivered to the nose becomes the same as the flow rate inhaled from the nose, and this dramatic reduction in flow rate results in a drastic reduction in the pump and battery capacity.

#### Precise Pressure Control by Fluid Modeling

Fluid modeling of PAPRs should be considered. For the PAPR prototypes (simple PAPR and controller PAPR) shown in Figure 1, the air supply through a nonwoven fabric filter is realized by a pump, and the exhaust through a nonwoven filter is created through the positive internal pressure. A simple modeling for these PAPRs is as follows.

#### Air Supply Flow Rate

The flow rate Q_in_ (ΔP, V) through the filter is determined by the pressure difference ΔP_f_ before and after the filter. The flow rate through the pump is determined by the pressure difference ΔP_p_ before and after the pump and the applied voltage (V) of the pump. When the differential pressure ΔP (=ΔP_f_ + ΔP_p_) inside and outside the PAPR and the pump applied voltage (V) are determined, the air supply flow rate Q_in_ is determined.

#### Exhaust Flow Rate

The flow rate Q_out_ (ΔP) through the filter is determined by the pressure difference ΔP_f_ before and after the filter.

#### Respiratory Flow

The flow difference Q_diff_ between the air supply flow rate Q_in_ and the exhaust flow rate Q_out_ can be expressed as follows.

Q_diff_ = Q_in_ (ΔP, V) – Q_out_ = Q_breath_ + Q_leak_ + Q_volume_

Here, Q_breath_=respiratory flow rate of the wearer of PAPR, positive with inspiration; Q_leak_=leak flow rate, positive for leakage from the inside to the outside; and Q_volume_=volume change inside PAPR, positive with volume increase.

Since the time averages of respiratory flow Q_breath_ and volume change Q_volume_ are zero, the time average of Q_diff_ is the time average of Q_leak_. In addition, Q_leak_ is expressed as a function Q_leak_ (ΔP) of the differential pressure ΔP, assuming that the shape of the gap between the face and the mask is constant. Furthermore, the volume change Q_volume_ is considered to be expressed as a function Q_volume_ (ΔP) of the differential pressure ΔP.

Q_breath_ = Q_diff_ – Q_leak_ (ΔP) – Q_volume_ (ΔP)

In this case, Q_breath_<0 is judged as expiration, and Q_breath_>0 is judged as inspiration. In this way, the exhalation and inhalation movements of the wearer can be detected in real time. For example, based on this detection result, the following control can be considered.

1. If an exhalation movement is detected, the minimum positive pressure setting (eg, 10 Pa) is set to minimize the resistance to exhalation movement while preventing leakage from the gap.

2. If an inhalation movement is detected, a strong positive pressure setting (eg, 100 Pa) is used to positively assist the inhalation movement.

In addition to the forced air supply by the pump and filter, the introduction of forced exhaust by the pump and filter enables the following differential pressure control.

1. When an exhalation movement is detected, a strong negative pressure setting (eg, –100 Pa) is used to actively assist the exhalation movement.

2. When an inhalation movement is detected, a strong positive pressure setting (eg, 100 Pa) is used to actively assist the inhalation movement.

In light of this, a PAPR facilitates the easy movement of the wearer’s exhalation and inhalation. In this case, the direction of leak flow at the possible gap is opposite to normal—from outside to inside during exhalation and from inside to outside during inhalation. It will be possible to detect coughing from the measurement results of the differential pressure ΔP. It will also be possible to estimate the possibility of infection of the wearer together with other measurement results such as body temperature. The ability to efficiently identify infected persons will enable efficient isolation and treatment of infected persons and will have a significant effect in reducing the spread of infection throughout the society.

#### Improved Design

All the 3 types of PAPRs shown in Figure 1 have bulky and exaggerated designs. As a lockdown alternative, the design may not be very important; however, it is better to have an excellent design. If the above flow path optimization achieves a dramatic reduction in the air supply flow rate, then a dramatic reduction in pump and battery size and various designs will become possible. Once PAPRs are widely accepted as a lockdown alternative, many people will be dissatisfied with the bare-bones PAPRs provided by the government; it is conceivable that companies of various genres will develop models with different characteristics.

#### Maintenance Method

It is necessary for every citizen to be able to easily perform maintenance such as cleaning and disinfecting the PAPR and replacing the nonwoven filter unit at home. However, when PAPRs are used as an alternative to lockdown, it is expected that the virus concentration in the external environment will be extremely low compared to the environment assumed in medical PAPRs due to the following reasons.

1. Infected persons would wear PAPRs.

2. PAPR has the ability to not only stop the entry of droplets and aerosols containing viruses but also prevent their release to the outside.

3. PAPR purifies indoor air in the same way as an air purifier.

Therefore, in terms of maintenance standards, it may be possible to set relatively lenient standards for nonmedical use PAPRs compared to those set for medical PAPRs, which are assumed to be used in environments with high virus concentrations such as hospital wards where infected people are congregated.

#### Variations Suitable for Different Places of Use and Activity Contents

The following variations should be developed, which are fine-tuned to suit various places of use and activities, as well as to suit societies and populations at different stages of social, economic, and cultural development: (1) a model that pursues comfort for everyday use; (2) models suitable for specific activities such as sports, eating, and drinking, for example, a model for eating and drinking with a face shield opening and closing mechanism and an air shower function or a model for jogging with a structure that mechanically separates the nose (exhalation) and mouth (inhalation); (3) a booth type model that wraps around a desk and a chair in an office, vehicle, restaurant, etc; (4) a model compatible with the standard unit of the ceiling-mounted air conditioner; and (5) a very inexpensive model suitable for low-income countries and regions.

#### PAPR With Information Terminal Function

The PAPR prototype (controller type) shown in Figure 1 is an all-in-one type PAPR with a computer and a power supply on the wearer’s head. Therefore, it is easy to make the PAPR an advanced information terminal by means of installing a computer equivalent to that of a high-end smartphone and adding various devices as follows: (1) equipped with smartphone function and virtual reality screen, (2) equipped with a noncontact input system using eye gaze and brain waves, and (3) equipped with a physical condition measurement and management system using body temperature sensors, cough sensors (pressure sensors), electroencephalogram sensors, etc. If the PAPR is comfortable to breathe, comfortable to wear, and has advanced information terminal functions, it is expected that some people will not be able to part with it. In particular, if a physical condition measurement and management system is installed to accurately estimate the presence or absence of infection, it will be easier to isolate, examine, and treat those who are deemed to have a high probability of being infected. In many cases, the various behavioral and activity restrictions during lockdown are uniformly applied to all persons under conditions where it is not known who is infected. If it becomes possible to know with a high degree of certainty who is infected, the use of PAPR as an alternative to lockdown can be changed to a more targeted approach.

#### Evaluation Indicators and Evaluation Methods

As an evaluation indicator of PAPR as a lockdown alternative, it is desirable to be able to quantitatively evaluate the effect of reducing the aforementioned β by means of wearing a PAPR. However, in order to estimate the β reduction rate with high accuracy, it is necessary to conduct elemental experiments and social experiments under various conditions.

The most important evaluation indicators of the basic performance of PAPR that should be obtained from elementary experiments are as follows: (1) reduction rate of virus-containing aerosols and droplets inhaled by potentially infectious persons wearing PAPR and (2) reduction rate of virus-containing aerosols and droplets exhaled by infected persons wearing PAPR. Current standards (eg, APF) usually refer only to the reduction rate of virus-containing aerosols and droplets inhaled by potentially infectious persons wearing PAPR. However, both (1) and (2) are considered to be equally important when requiring the uniform wearing of PAPRs by the general public in cases where presence or absence of infection is unclear for the purpose of reducing the effective reproduction number R_t_. In the 3 PAPR models shown in Figure 1, positive pressure was used to prevent outside air from entering directly through gaps, with an emphasis on protecting the inside (wearer) from the outside. If the above (1) and (2) are equally important, then it is equally important to protect the wearer from the outside environment and to protect the people from the wearer, thereby indicating the it is not necessary to make the internal pressure positive.

#### Impact of the PAPR Internal Environment on the Mind and Body

If PAPR is considered as an alternative to lockdown measures, the impact of the PAPR internal environment on the mind and body of the wearer will become important. It is necessary to comprehensively investigate the relationship between the following 2 types of parameters from the viewpoint of the influence of the PAPR internal environment on the mind and body of the wearer.

### Physical Parameters Related to the PAPR Internal Environment

The physical parameters to be considered are concentrations of particulate pollutants (droplets, aerosols, pollen, particulate matter 2.5, mite corpses, dust, etc), gaseous pollutants, gas composition (carbon dioxide concentration, oxygen concentration, etc), differential pressure, temperature, humidity, acoustic characteristics (sound transfer characteristics, noise, etc), vibration, and airflow.

### Biological and Psychological Parameters of PAPR Wearers

The biological and psychological parameters are respiratory status, electroencephalogram, body temperature, pulse rate, comfort, safety (physical danger, probability of infection), and degree of relaxation.

#### Balance Between Infection Control Efficiency and Personal Freedom Through the Use of IoT

**Figure 5:**
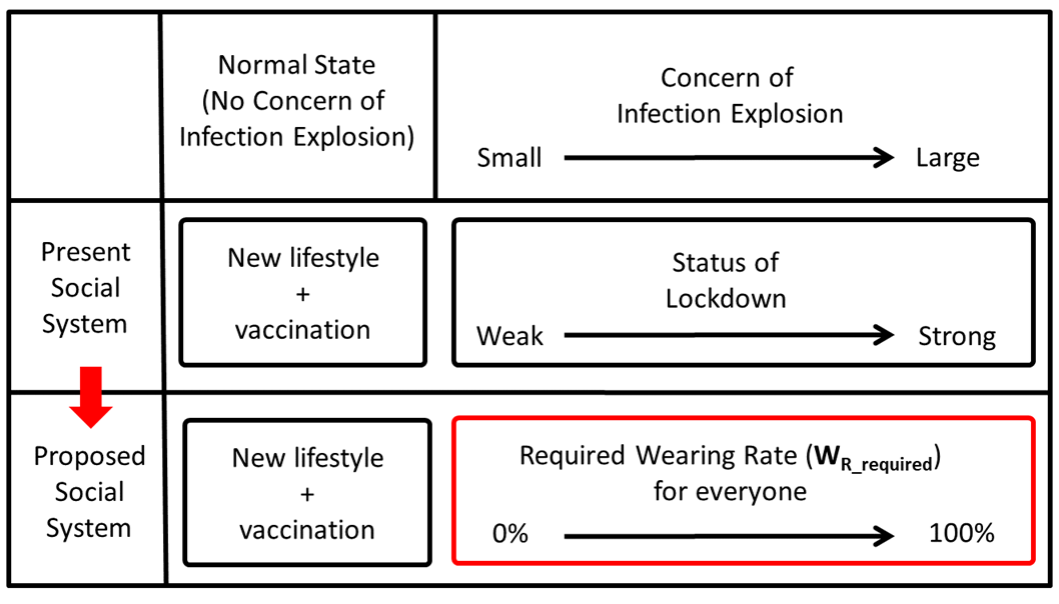
The social system with the powered air purifying respirator wearing rate network management system.

As shown in Figure 5, the operation of the PAPR wearing rate network management system led by the government will be performed as follows.

1. The government will distribute PAPRs (with smartphone connectivity) with sufficient aerosol shielding performance to all citizens as emergency equipment. Each citizen installs an app with a wearing rate proof function on his/her own smartphone.

2. If the estimated effective reproduction number R_t_ is high and there is concern about an outbreak of infection, the government will (1) set the target effective reproduction number R_t_target_, (2) solve the formula based on appropriate assumptions to calculate the required wearing rate W_R_required_ required to achieve R_t_target_, (3) show W_R_required_ and require all citizens to comply with it—each citizen can spend their time without PAPR at any place (party venue, restaurant, pub, etc) and any time by showing proof of wearing rate W_R_ within the scope of fulfilling their obligations, and (4) pay close attention to changes in the effective reproduction number R_t_ and raise W_R_required_ if the goal of controlling the spread of infection is in jeopardy. Conversely, if it exceeds the target, lower W_R_required_ and increase the degree of freedom in citizen life.

If the PAPR lockdown alternatives are strong enough, the government can quickly contain the spread of infection by setting W_R_required_ to 1.0 (100%) even when the government makes a big mistake in estimating W_R_required_ and falls into the worst situation. In that case, the government can conduct various trials and countermeasures on various assumptions and hypotheses with a leeway. The government is freed from constraints that limit them to overly conservative measures. In addition, throughout the entire process, the government will be able to improve the accuracy of the above formula based on appropriate assumptions by using the big data collected on the relationships between “changes in aerosol shielding performance of PAPR (air intake side, exhaust side), PAPR wearing rate W_R_, and wearing condition, etc” and “changes in infection spread status and the effective reproduction number R_t_.”

## Discussion

### Principal Findings

In this study, the feasibility of the following 2 ideas was examined. First, the construction of a social system using PAPR with similar infection control ability as lockdown measures and with less economic and social damage as an alternative to lockdown is possible. Second, balancing the efficiency of the government’s infection control and each citizen’s personal freedom is possible by means of an IoT system.

### Extended Functionality and Privacy Protection in the PAPR Wearing Rate Network Management System

By utilizing PAPR with several sensors (thermometer, cough sensor, etc), the government can make this system much more powerful than conventional apps for measuring contact with infected persons. For example, the system may be able to improve the accuracy of infection detection based on big data concerning changes in body temperature, cough (condition and frequency), and the presence or absence of severe disease after infection until the onset of illness. In the case of PAPR equipped with both air supply and exhaust pumps, the wearer can switch the internal pressure between positive pressure when not infected and negative pressure when infected, thus prioritizing the prevention of the spread of infection in the society as a whole. From the viewpoint of privacy protection, social discussion is necessary for the following matters: (1) how much of the information from PAPRs should be passed on to the government server? and (2) how should the government’s use of personal information be curbed? Especially for (2), it is considered necessary to develop and construct a technical and social mechanism to realize a brake. In order to prevent misuse of personal information, it is conceivable to apply the proposal for a street camera system’s perfect recording of usage history by a reliable third party as the first step [30]. Methods to substitute each restriction in lockdown with PAPR utilization need to be considered in various social systems. Those who wish to use PAPR as a substitute for the constraints imposed by lockdown need to prepare to obtain the PAPR before the pandemic. Therefore, it is also important for the society as a whole to ensure and disseminate information on how to obtain PAPRs.

Different countries have different governance systems. In some countries, it might not be easy to make the public understand that PAPR can be used an alternative to lockdown measures—they may make it an option and not a mandate. This paper discusses how PAPR can substitute the primary constraints imposed by lockdown. Even in cases of other alternatives such as combination of lockdown and free mobility of low-risk populations during COVID-19 [32], PAPRs may be used for controlling the infection rates. The proposed PAPR wearing rate network management system utilizes IoT technology, which is currently being widely pursued by various societies and companies. In order to build a social system that makes the government’s control of the spread of infection more efficient and that respects the freedom of individuals to the maximum extent, social experiments should be first conducted under various conditions to identify the challenges and improve the effectiveness of PAPRs.

### Society of People Breathing Purified Air

Further, although this is a discussion that is far from the main point of this paper, if truly high-performance, comfortable, and low-cost PAPRs are successfully developed through this research and subsequent research and developments, it is possible that many people will desire PAPR-purified air instead of the air around them. This is similar to the situation of drinking water, that is, just as how populations consume purified water through water-treatment and water-purification technology rather than water from ponds and rivers, people would prefer purified air to breathe. It can be expected that many citizens will wear PAPRs when they go out, regardless of whether the government asks them to do so. As more people breathe purified air, there may be concerns about the public’s immune system being weakened against airborne diseases and pollen allergies. However, there is no dispute that water-borne infectious diseases have become controllable because many people drink only purified water, and it would not be advisable to drink water without purification, as was the case in primitive times. A society in which the majority of the population breathes purified air will be resilient to all airborne diseases. The construction of such a society has the potential to be an opportunity for a historic change in the human race, which has been plagued by airborne diseases.

### Conclusions

This study examines the feasibility of 2 ideas. First, this study shows that it is possible to construct a social system using PAPR with similar infection control effects as lockdown measures and with less economic and social damage as a means of temporarily reducing the effective reproduction number R_t_. Second, the PAPR wearing rate network management system balances the achievement of the efficiency of the government’s infection control and each citizen’s personal right to choose the time and opportunity not to wear PAPR during a pandemic.

## Abbreviations

APF: assigned protection factor
IoT: Internet of Things
PAPR: powered air purifying respirator

## Data Availability

This study was conducted using deductive thinking based on publicly available information. No confidential data were used in this study.
